# The *Escherichia coli* SOS Gene *dinF* Protects against Oxidative Stress and Bile Salts

**DOI:** 10.1371/journal.pone.0034791

**Published:** 2012-04-16

**Authors:** Jerónimo Rodríguez-Beltrán, Alexandro Rodríguez-Rojas, Javier R. Guelfo, Alejandro Couce, Jesús Blázquez

**Affiliations:** Centro Nacional de Biotecnología (CSIC), Madrid, Spain; The Scripps Research Institute, United States of America

## Abstract

DNA is constantly damaged by physical and chemical factors, including reactive oxygen species (ROS), such as superoxide radical (O_2_^−^), hydrogen peroxide (H_2_O_2_) and hydroxyl radical (•OH). Specific mechanisms to protect and repair DNA lesions produced by ROS have been developed in living beings. In *Escherichia coli* the SOS system, an inducible response activated to rescue cells from severe DNA damage, is a network that regulates the expression of more than 40 genes in response to this damage, many of them playing important roles in DNA damage tolerance mechanisms. Although the function of most of these genes has been elucidated, the activity of some others, such as *dinF*, remains unknown. The DinF deduced polypeptide sequence shows a high homology with membrane proteins of the multidrug and toxic compound extrusion (MATE) family. We describe here that expression of *dinF* protects against bile salts, probably by decreasing the effects of ROS, which is consistent with the observed decrease in H_2_O_2_-killing and protein carbonylation. These results, together with its ability to decrease the level of intracellular ROS, suggests that DinF can detoxify, either direct or indirectly, oxidizing molecules that can damage DNA and proteins from both the bacterial metabolism and the environment. Although the exact mechanism of DinF activity remains to be identified, we describe for the first time a role for *dinF*.

## Introduction

Oxidative stress, the inevitable consequence of living in an oxygen-rich environment, occurs when the cellular redox balance is upset by increased doses of reactive oxygen species (ROS). Microorganisms living in aerobic environments are constantly exposed to ROS, which are generated by the aerobic metabolism and environmental agents. ROS, including superoxide radical (O_2_^−^), hydrogen peroxide (H_2_O_2_) and hydroxyl radical (•OH), are highly reactive molecules that can damage key cellular components, including DNA, proteins, carbohydrates and lipids. To defend themselves against ROS injuries, microorganisms have developed different constitutive and inducible mechanisms, including scavenging systems, like superoxide dismutases (SOD) and catalases/peroxidases, export of redox-cycling substances, like the AcrAB-TolC efflux pump, and DNA repair systems, like DNA-glycosilases [1]. In addition, commensal and pathogenic bacteria have to cope with oxidative responses from the host, such as bile salts in the gastrointestinal tract or H_2_O_2_ from phagocytes.

Specific mechanisms to protect and repair DNA lesions produced by reactive forms of oxygen have been developed. When DNA lesions are persistent the SOS system, an inducible response, is activated to rescue cells from severe DNA-damage [1]. In *Escherichia coli* the expression of more than 40 genes [2], many of them playing key roles in DNA damage tolerance mechanisms, is regulated by the LexA repressor [3], which autogenously regulates its own transcription [4]. When no DNA damage occurs, the cellular levels of LexA repressor suffice to repress the system. The blockage of DNA replication originated by DNA damage, including that produced by ROS, generates stalled replication forks and, consequently, single stranded DNA (ssDNA) [1]. This ssDNA is the molecular distress signal allowing the nucleation of RecA monomer protein around it. The interaction ssDNA-RecA produces the RecA* coprotease activity, which promotes the autocleavage of the LexA repressor. This process decreases the intracellular level of LexA, which in turn releases the repression of SOS genes, switching on the system. DNA repair functions, such as excision repair (UvrABC), Holliday resolution junctions (RuvAB), and translesion synthesis (TLS) polymerases, are SOS-induced [1]. Finally, when the distress signal disappears, the level of RecA* decreases and that of LexA repressor increases, leading the SOS system to the repressed state.

By generating random Mu*d*1-*lacZ* transcriptional fusions Kenyon and Walker [5] identified a set of damage inducible (*din*) genes whose expression was increased by different SOS-inducing treatments. The function of many of these *din*, and other SOS, genes has been identified (for a review see [1]). However, the role of some, such as *dinF*, remains unknown. By sequence homology it has been deduced that *dinF* encodes a 49 kDa multidrug and toxic compound extrusion (MATE) family membrane protein [6], [7]. Members of the MATE family of transporters characteristically possess 12 putative transmembrane domains and have been found in all three domains of life, including humans, where they mediate the efflux of organic cations using the transmembrane proton gradient as a driving force [6], [7].

Recently, we have shown that expression of the *E. coli* MATE protein NorM protects the cells from the H_2_O_2_ killing effect, particularly when other protective mechanisms are absent [8]. These results led us to test whether the expression of DinF could also diminish the H_2_O_2_ lethality. We have also analyzed different effects (intracellular ROS levels, protein carbonylation, antibiotic resistance, and mutation rate) produced by the expression of *dinF* in different genetic backgrounds. Because in *E. coli dinF* forms an operon with *lexA,* the master regulator of the SOS response, we have studied the putative co-regulation of *lexA* and *dinF* transcription in all sequenced bacterial genomes. Finally, as both genes appear to form a single operon only in *Enterobacteria*, we have analyzed whether *dinF* protects from bile salts, a known oxidant product present in the gastrointestinal tract [9].

## Materials and Methods

### Bacterial strains and plasmids

The *E. coli* strain NR10831 [F′CC101] (*ara, thi, rif* ^R^, *nal* ^R^, *metB,* Δ*prolac*) was a gift from I. Fijalkowska (Institute of Biochemistry and Biophysics, Warsaw, Poland). The F′CC101 carries an specific *lacZ* mutation affecting residue Glu-461 in β-galactosidase. Only an AT to CG base substitution will restore the glutamic acid codon and the Lac^+^ phenotype [10]. The strains NR10831 Δ*dinF* and NR10831 Δ*mutT* were constructed by P1 transduction of the Δ*dinF*::Kan and Δ*mutT*::Kan alleles from the corresponding strains of the Keio Collection [11] as described [12]. Because of NR10831 is resistant to rifampicin, the *E. coli* BW25113 strain and its mutant derivatives (also constructed by P1 transduction) were used to estimate mutation rates to rifampicin resistance. To discard that the *dinF* deletion could alter the *lexA* regulation/expression and that of other *lexA*-regulated genes, the expression of both *recA*::GFP and *lexA*::GFP transcriptional fusions [13] were studied under SOS induced and non-induced conditions. No differences were found between wild-type and *dinF*-defficient strains (data not shown).

The vector pCA24N [14] and its derivative pDinF, containing the wild-type *dinF* gene, were obtained from the Complete Set of *E. coli* K-12 Open Reading Frame Archive (ASKA) library [14].

### Materials and media

The bacteria were grown in liquid M9 minimal medium with 1% glucose and a mixture of amino acids (10 µg/ml each) or LB. The following materials were obtained from Sigma: IPTG (isopropyl-β-D-thiogalactopyranoside), chloramphenicol, ripampicin, trizma-base, sodium dodecyl sulphate (SDS), DNAse, RNAse, norfloxacin, ofloxacin, streptomycin, mitomycin C and ethidium bromide. We also used the following materials: lysozyme and glycerol (United States Biochemical Corporation), ampicillin (Biochemie GmbH) and bile salts (N° 3, Pronadisa, Spain), ciprofloxacin and gentamicin (Normon SA, Spain), ceftazidime (Combino Pharm), kanamycin (Q-biogene, USA) and H_2_O_2_ (FMC Foret, Spain). Carbonylated proteins were detected using the chemical and immunological reagents from the OxyBlot Oxidized Protein Detection Kit (Chemicon). Dihydrorhodamine 123 (DHR) for detection of ROS was from Enzo® Life Sciences.

### Estimation of H_2_O_2_-induced cell death

Strains were grown at 37°C in M9 supplemented with appropriate antibiotics to mid-exponential phase and washed with 0.9% NaCl solution. Cells were treated with different concentrations of H_2_O_2_ (1, 12.5, 25 and 50 mM) for 30 min at 37°C and washed with 1 ml of 0.9% NaCl. A non-treated control was also included. Appropriate dilutions were immediately plated onto LB plates and incubated overnight at 37°C to determine viability. Experiments consisted of five independent cultures for each strain. Cell survival was calculated by comparing the number of colony forming units (cfu) of treated to those of the untreated cells.

### Estimation of mutation rates

Lac reversion assays were carried out in M9 minimal medium. Inocula were grown with glucose as carbon source and plates were supplemented with lactose as unique carbon source according to Miller [12]. The scavenger strain MEC222 [15], harboring a truncated *lacZ* allele (*lacZ*ΔT::*cat*) with the c-terminal region replaced by the *cat* cassette, was added to the lactose agar MM before being spread (40 µl of a stationary phase culture per litre of media, approximately 10^7^ cells/plate). Plates were stored overnight at room temperature. The desired cultures for Lac reversion assays were spread onto a M9 top agar layer, without carbon source and supplemented with 5-bromo-4-chloro-3-indolyl-3-D-galactoside (X-Gal), as described by Miller [12].

To calculate the mutation rate, pre-inocula were initiated in tubes with 3 ml of M9 glucose directly from frozen samples. The pre-inocula were grown at 37°C overnight to the stationary phase. From each culture about 10^4^ cells were inoculated in 40 ml of M9 glucose and divided into 4 independent cultures, 10 ml each and less than 10^4^ cells/culture. These inocula were grown for 24 hours. Appropriate dilutions of the saturated cultures were plated onto selective medium Lac X-gal MM to determine the number of Lac^+^ mutants, respectively. LB plates were used to determine the total cfu. To calculate the mutation rate to rifampicin resistance, the strain BW25113 and its mutant derivatives were inoculated in LB with appropriate antibiotics directly from frozen samples and incubated overnight. Less than 10^4^ cells were inoculated in each one of 4 flasks containing 2 ml of LB and allowed to grow for 24 h at 37°C with strong shaking. Then, appropriate dilutions were plated onto LB-rifampicin (100 µg/ml) agar plates to select spontaneous resistant mutants. LB plates were used to estimate the number of viable cells in the culture. This protocol was performed by quadruplicate.

Mutation rates were calculated using the Ma-Sandri-Sarkar maximum likelihood (MSS-ML) method [16] as implemented by Falcor web tool [17], [18].

### Flow cytometry

Flow cytometry analysis was performed using the H_2_O_2_-activated fluorescent dye dihydrorhodamine 123 (DHR). DHR is a probe for detection of intracellular reactive oxygen species. It is oxidized into rhodamine 123 which produces a maximal emission at 529 nm when excited at 507 nm (Enzo® Life Sciences). Wild type and mutant derivatives were grown in M9-glucose at 37°C to mid exponential phase of growth. Cells (0.5 ml/culture) were pelleted by centrifugation, and resuspended in saline containing 15 µM DHR, and then incubated for 15 min and diluted 1∶500 in phosphate-buffered saline. The fluorescence levels (excitation 488 nm and emission 530 nm) of 15,000 cells were then counted for each strain under each condition using a FACSCalibur cytometer (BD Biosciences). WinMDI (The Scripps Institute, Purdue University, USA) was used for data analysis. Values obtained were the geometric mean of the fluorescence from the 15,000 cells. Experiments were performed three times.

### Determination of the cellular level of protein carbonylation

Wild type and mutant derivatives were grown overnight and then each one was split into two cultures (one control and one treated with 50 mM H_2_O_2_) and incubated as described above for 30 min, and cultures were submitted to peroxide challenge. After this time, peroxide was removed by centrifugation. Then, cells were washed, resuspended in M9 medium preheated to 37°C and further incubated. Cells were lysed as follows: 1 ml of the culture was washed with 50 mM Tris buffer (pH 7.5) and centrifuged for 10 min at 14,000 rpm. The pellet was re-suspended in 150 µl lysis buffer containing 0.5 mg/ml lysozyme, 20 µg/ml DNAse, 50 µg/ml RNAse, 1 mM EDTA, and 10 mM Tris (pH 8). 15 µl of 10% SDS solution was added and the cells were incubated at 100°C for 5 min. To examine the level of protein carbonylation in these lysates, we used the Chemicon OxyBlot kit to derivatize the carbonyl groups in the protein side chains to 2,4-dinitrophenylhydrazone (DNP-hydrazone) by reaction with 2,4-dinitrophenylhydrazine. These DNP derivative crude protein extracts were dot blotted onto a nitrocellulose membrane, which was incubated with primary antibody, specific to the DNP moiety of the proteins, and subsequently incubated with secondary (goat anti-rabbit) horseradish peroxidase-antibody conjugate directed against the primary antibody. Carbonylation was observed by ECL (Amersham Pharmacia Biotec). The intensity of each dot was quantified by densitometry analysis using the Image Master VPS-CL. The intensity of each dot was normalized to equal levels of protein, which were determined using Bradford reagent (Bio-Rad) and expressed in femtomoles of DNP, according to the control of the OxyBlot kit. Assays were done by triplicate.

### Determination of minimal inhibitory concentrations

Minimal inhibitory concentrations (MIC) of ciprofloxacin, norfloxacin, ofloxacin, ampicillin, ceftazidime, streptomycin, kanamycin, gentamicin, bile salts, H_2_O_2_, mitomycin C and ethidium bromide were determined for the strain NR10831 harbouring either the empty vector pCA24N or the plasmid expressing *dinF* (pDinF). MICs with these two strains were studied by adding IPTG (50 µM final concentration) to achieve maximal expression. MICs were determined by inoculating mid-log phase grown strains in the wells of a 96-microwell plate. The bacterial inoculum was prepared using the same procedure in all cases. Approximately 10^3^ cells from overnight cultures were inoculated into tubes containing 10 ml of LB broth supplemented with appropriate antibiotics and IPTG. The tubes were incubated at 37°C with strong agitation until the mid-log phase of growth (approximately 10^8^ cells/ml). Then, 2×10^4^ to 4×10^4^ cells from these cultures were inoculated into each microdilution well containing LB and doubling concentrations of the desired substance. Incubation was at 37°C for 24 h. The MIC was defined as the minimal concentration where no growth was observed. Four replicas were performed for each antibiotic and strain.

### Competitions in bile salts

After plating in M9 with lactose as unique carbon source spontaneous Ara+ revertants were isolated for the strains NR10831 and NR10831 *ΔdinF*::Kan and were transformed subsequently with plasmids pCA24N and pDinF. Competition assays were performed as described previously [19]. Briefly, 100 µl of a 1∶1 mixture of overnight cultures of each strain were inoculated into flasks containing 9.90 ml of fresh LB medium supplemented with different concentrations of bile salts (Bile salts n° 3, Pronadisa, Spain) and allowed to grow for 24 h at 37°C with strong shaking (250 rpm). In order to distinguish between strains, in all competitions one competitor was Ara+ and the other Ara-. Competitions were repeated reversing the marker (competitor one Ara+ and competitor two Ara- and vice versa) to ensure that the Ara mutation has no effect on fitness determination. Initial (N_0_) and final (N_t_) densities of each strain were estimated by plating appropriate dilutions on LB and M9 with lactose as unique carbon source agar plates. Relative fitness was calculated as the ratio of growth rates (r) of each strain or W = r_ΔdinF_/r_wt_ where r = ln N_t_/N_0_. Results given are the mean fitness of eight replicates.

### *lexA-dinF* operon in bacterial genomes

To study the putative co-regulation of *lexA* and *dinF*, we performed a search in operonDB (http://operondb.cbcb.umd.edu) for the *E. coli* MG1655 genome. Then, we selected the *lexA-dinF* operon and analysed the co-occurrence of that operon in all bacterial genomes stored in the database.

## Results

### Search of NorM homologues in *E. coli*

**Figure 1:**
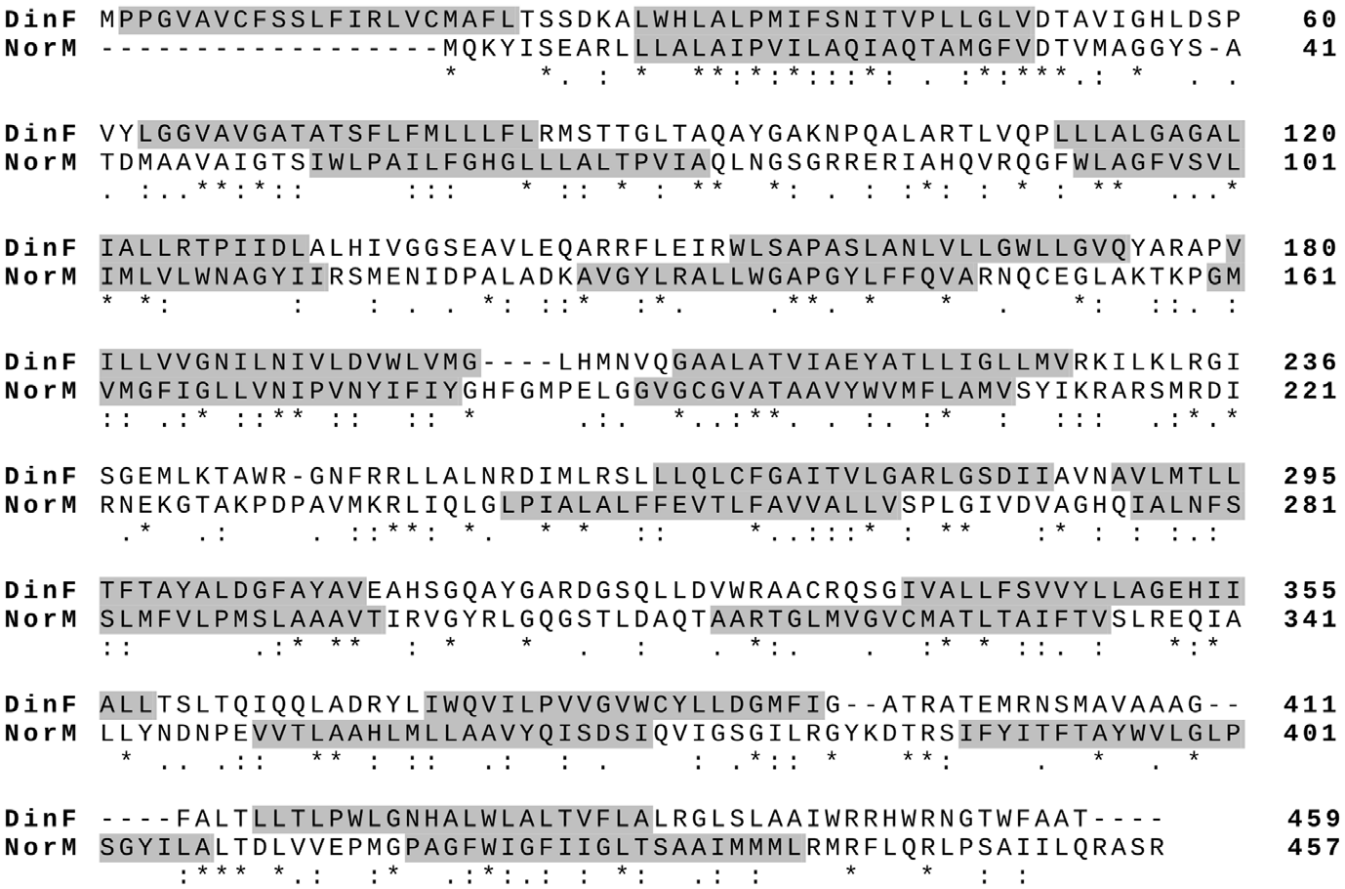
Sequence alignment of DinF and NorM. Aligment (ClustalW) of sequences from E. coli K12 was done according to Uniprot tools (http://www.uniprot.org) [42]. Predicted transmembrane domains are highlighted in grey.

DinF and NorM from *E. coli* K12 show a 20% identity and a 36% similarity [20] according to a Blast search done at the NCBI site (http://ncbi.ace.uk) (Fig. 1). DinF contains, like NorM, twelve predicted transmembrane domains (Fig. 1). Therefore, in principle, a similar activity can be expected for both of them.

### H_2_O_2_-induced killing

**Figure 2:**
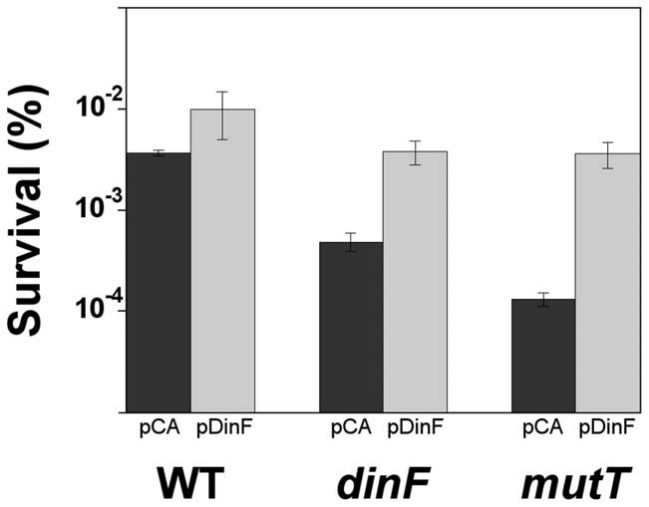
Viability after H_2_O_2_ treatment. The data represent survival percentages after 30 min of 50 mM H_2_O_2_ treatment. Shown are the strains NR10831 (WT) and its mutant derivatives Δ*dinF* and Δ*mutT* harboring the empty vector pCA24N (black) or the plasmid expressing *dinF*, pDinF (gray). The error bars indicate the standard deviation of four independent replicates.

Previous results from our laboratory showed that the expression of *norM* reduced the H_2_O_2_-induced killing [8]. Consequently, we tested whether the expression of *dinF* could also decrease the killing by H_2_O_2_. Figure 2 shows that sensitivity to H_2_O_2_-mediated killing of cells lacking *dinF* (Δ*dinF*::Kan) is clearly higher than that of the wild type cells. The multicopy expression of *dinF* (pDinF) rescues the wild type survival level. To know how much dinF is over-expressed in the plasmid, we performed RT-qPCR of *dinF* in both NR10831 and NR10831 Δ*dinF*::Kan (pDinF). Expression of *gapA*, which encodes the GAPDH (glyceraldehyde-3-phosphate dehydrogenase) enzyme, was used as an endogenous reference [21]. The results show that *dinF* expression is about 18 times higher in the plasmid than in the chromosome (data not shown).

### Expression of *dinF* protects from H_2_O_2_-induced killing in the absence of MutT activity

Oxidation of guanine to 7,8-dihydro-8-oxo-guanine (8-oxoGTP) is especially noteworthy because it is highly mutagenic. *E. coli* possesses an efficient system to reduce the mutagenic effects of 8-oxoG, the GO repair system, consisting in three proteins, MutM, MutY and MutT [22]. MutT is a nucleoside triphosphate pyrophosphohydrolase, which converts 8-oxodGTP to 8-oxodGMP and pyrophosphate, inactivating this mutagenic activity. In the absence of MutT there is an increase in AT to CG mutations [22].

GO-deficient cells have been demonstrated to be more sensitive to H_2_O_2_-induced killing than those of the wild-type [8], [23], [24] *via* a still unknown mechanism. Interestingly, multicopy expression of *norM* was able to increase survival of *mutT*-deficient cells after exposure to H_2_O_2_ [8]. Figure 2 shows that multicopy expression of *dinF* promotes protection of *mutT*-deficient cells from H_2_O_2_-induced killing.

Overall, all these results demonstrate that DinF protects cells from H_2_O_2_-induced killing, mainly in the absence of *dinF* or *mutT*.

### Effect of *dinF* expression on intracellular ROS levels

**Figure 3:**
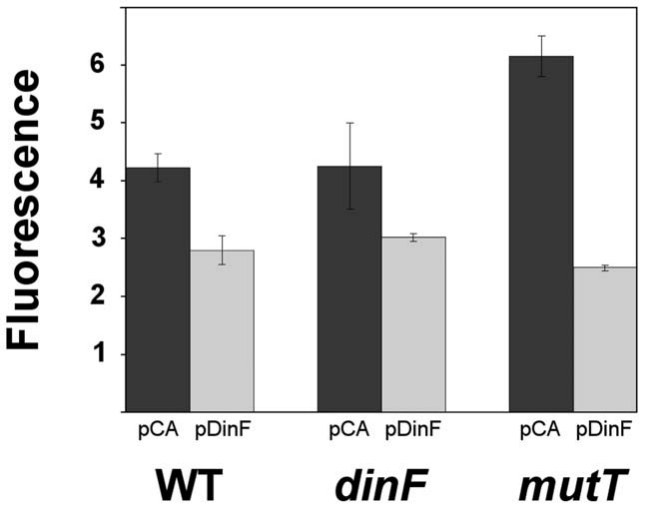
ROS levels in the *E. coli* wild type, *dinF* and *mutT* derivatives. Strains harboring either the empty vector pCA24N (black) or the *dinF*-containing plasmid pDinF (gray) treated with DHR. Data represent the mean values of three independent measurements of the spontaneous fluorescence of 15,000 cells as measured by flow cytometry. The error bars indicate the standard deviation of three independent replicates.

The above results suggest that DinF may control the level of intracellular ROS, which cause H_2_O_2_-induced killing. If this is true, the intracellular ROS levels should be diminished upon *dinF* expression. To assess this, the intracellular ROS levels in dihydrorhodamine 123 (DHR)-treated cells by flow cytometry were studied. DHR is a probe for the detection of intracellular reactive oxygen species. It is oxidized into rhodamine 123, which produces a maximal emission at 529 nm when excited at 507 nm (Enzo® Life Sciences). Figure 3 shows that the expression of *dinF* in the multicopy plasmid pDinF produces a slight but consistent decrease in the amount of intracellular ROS in both the wild type and Δ*dinF* strains. Figure 3 also shows that *dinF* expression produced a great decrease in intracellular ROS when expressed in a *mutT* background.

### Effect of *dinF* expression on protein carbonylation

**Figure 4:**
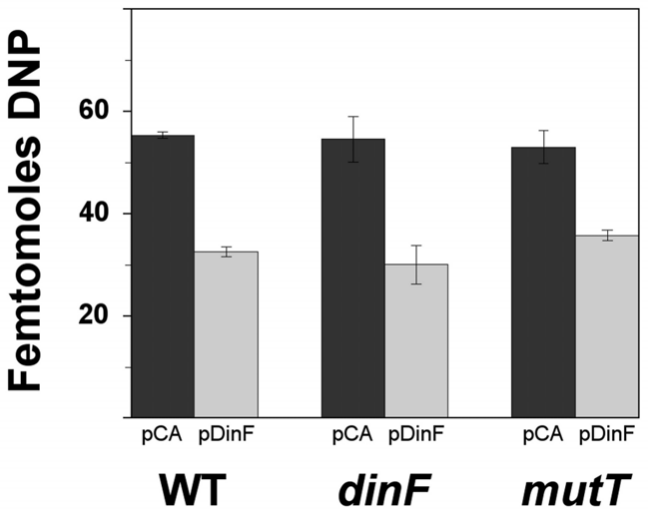
Protein carbonylation. Bar graph quantitating the protein carbonylation (femtomoles of DNP) in cells harboring the empty vector pCA24N (black) or the *dinF*-containing plasmid pDinF (gray) in the wild type, Δ*dinF* and Δ*mutT* derivative strains, following treatment with 10 mM H_2_O_2_ for 15 min. The data are the mean values from four separate experiments and error bars represent the standard deviation.

Carbonyl groups are introduced into protein side chains by site-specific oxidative modifications. Thus, carbonyl quantification provides an estimation of the oxidation status of proteins. The effect of *dinF* on protein carbonylation was studied in non-treated cells. The level of spontaneous protein carbonylation in the wild type, Δ*mutT* and Δ*dinF* strains growing in exponential phase was undetectable with the OxyBlot kit. However, when submitted to H_2_O_2_ pre-treatment, as indicated in the experimental procedures section, the expression of *dinF* in the multicopy plasmid pDinF produced a clear decrease in the amount of carbonylated proteins in wild type, Δ*dinF* and Δ*mutT* strains (Fig. 4).

### Effect of *dinF* expression on mutation rate

**Figure 5:**
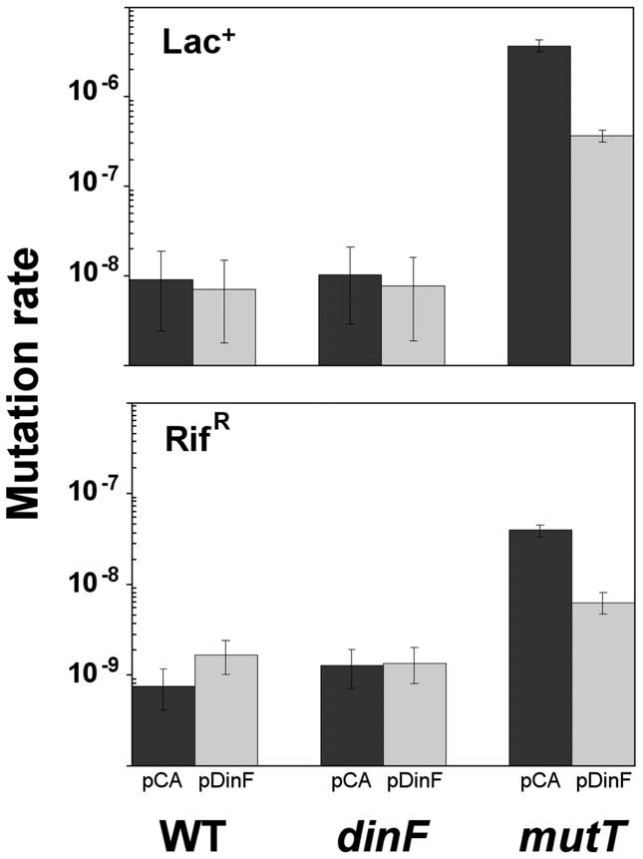
Effect of *dinF* expression on spontaneous mutation rate. The upper graph shows the mutation rate (expressed as mutations/cell/generation) of the Lac^−^ to Lac^+^ reversion for the wild type NR10831 (F′CC101) and its mutant derivatives, Δ*dinF* and Δ*mutT* harboring either the empty vector pCA24N (black) or the plasmid expressing *dinF,* pDinF (gray). The lower plot shows the mutation rate to rifampicin resistance of the wild type BW25113 and its mutant derivatives Δ*dinF* and Δ*mutT* harboring the same plasmids. Values were calculated by the MMS-ML method. Error bars represent 95% confidence intervals.

Lac reversion assays were carried out with the strain NR10831 (F′CC101) and its mutant derivatives. The lac assay detects the very specific mutational spectrum of the *mutT* allele (AT to CG) changes in the F′CC101 episome, produced by increased levels 8-oxodGTP in the cell. To further confirm the mutation rates with a different marker, mutation rates to rifampicin resistance were performed. Because NR10831 (F′CC101) is resistant to rifampicin, BW25113 and its mutant derivatives were used. Figure 5 shows that plasmid expression of *dinF* decreased significantly (two tailed Student's t-test; p<0.05 in both cases) mutation rate in the Δ*mutT* strains. The decrease was moderate (ten and six-fold for lactose reversion and rifampicin resistance, respectively), suggesting that expression of *dinF* can not cope with the high number of 8-oxodGTP molecules generated in the *mutT* background.

### *lexA-dinF* operon in other species

In *E. coli* the *dinF* gene is located a few base pairs after *lexA* (the master repressor of the SOS system) and seems to form a transcriptional unit with it [5], thus suggesting an strict control of *dinF* transcription by the LexA repressor and, consequently an important role for *dinF* in alleviating DNA-damage. In order to investigate whether this co-regulation is maintained in other species, we used operonDB database, which analyzes the co-occurrence of homologous genes together in the same direction and strand in different bacterial sequenced genomes [25]. We found that the *lexA-dinF* operon is maintained in 77 bacterial genomes from those stored in operonDB, including gammproteobacteria from the *Enterobacteriaceae* family (*Citrobacter, Salmonella, Klebsiella, Escherichia* and *Shigella*) and from the *Vibrionaceae* family (*Vibrio cholera, V. fischeri, V. harveyi, V. parahaemoliticus* and *V. splendidus*). Thus, the particular gene order of the *lexA-dinF* operon is maintained in bacteria living in the gastrointestinal tract.

### Effect of *dinF* on protection from bile salts killing

**Figure 6:**
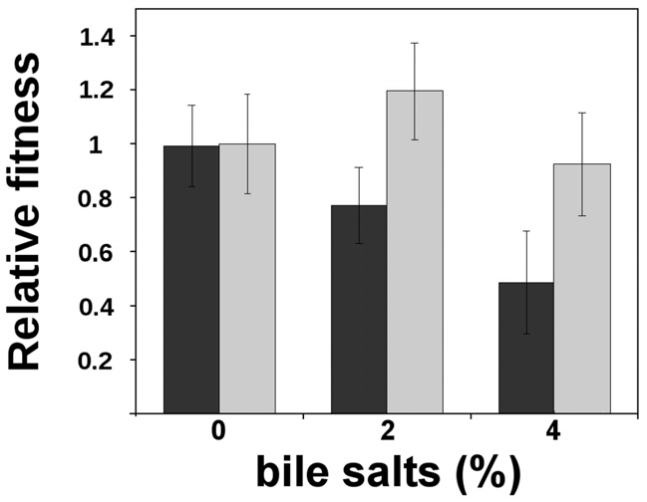
Fitness under bile salts challenge. Black bars represent the mean results of competition experiments between the NR10831 Δ*dinF ara^+^* (pCA24N) and its parental wild type strain NR10831 *ara^−^* (pCA24N), and viceversa. Grey bars represent the mean results of competition experiment of strain NR10831Δ*dinF* (pDinF) *vs* NR10831 (pCA24N) in order to see the effect of the complemented mutant. Error bars represent 95% confidence intervals around the mean.

Because both *lexA* and *dinF* genes appear to form a single operon only in *Enterobacteria*, we have analyzed whether *dinF* protects from bile salts, a known oxidant product present in the gastrointestinal tract [9]. Table 1 shows that expression of *dinF* in the wild type strain slightly increases MIC of bile salts. To further verify this effect, we performed competition assays between the strains NR10831 Δ*dinF* (pCA24N) and its parental wild type NR10831 (pCA24N) in concentrations of bile salts ranging from 0% to 4%. Figure 6 shows that in the presence of 2% and 4% bile salts, the absence of *dinF* implied a fitness cost of 20% and 50%, respectively (black bars). In order to assess the effect of the complementation of the mutant strain with the plasmid expressing *dinF*, competitions between strains NR10831 Δ*dinF* (pDinF) and wild type NR10831 (pCA24N) were performed. In the same figure 6, grey bars represent the results from these competitions. When pDinF was used to complement the *dinF* deletion, the mutant strain recovered a fitness value similar to that of the wild type strain, having a significant higher fitness (two tailed Student's t-test, p<0.05 in both cases). Thus, DinF contributes to final fitness when cells grow in presence of bile salts.

**Table 1:** MIC Results.

Product	WT pCA24N 50 µM IPTG	WT pDinF 50 µM IPTG
Ampicillin	16^a^	16
Ceftazidime	0.125	0.125
Streptomycin	8	8
Kanamycin	8	8
Gentamicin	1	1
Ciprofloxacin	0.0625	0.0625
Norfloxacin	1	1
Ofloxacin	0.5	0.5
**Bile Salts**	**6.25%**	**12.5%**
H_2_O_2_	1 mM	1 mM
Mitomycin C	4	4
Ethidium Bromide	62.5	62.5

a Unless otherwise specified concentrations are expressed in µg/ml.

Bold: Differences found only for this compound.

### Effect of *dinF* expression on toxic compounds protection

The proteins belonging to the same MATE family, have been described as multidrug efflux pumps able to confer resistance to several antimicrobial agents, such as norfloxacin, ethidium bromide (EtBr), and some aminoglycosides, via a mechanism requiring the proton motive force [26]. In addition, the expression of *dinF* from *Ralstonia solanacearum* in *E. coli* conferred resistance to several toxics, including ampicillin, acriflavine and ethidium bromide [27]. Consequently, expression of the *E. coli dinF* gene in *E. coli* may also confer resistance to some drugs. MICs of ciprofloxacin, norfloxacin, ofloxacin, ampicillin, ceftazidime, streptomycin, kanamycin, gentamycin, bile salts, H_2_O_2_, mitomycin C and ethidium bromide were determined for the strain Δ*dinF* harbouring either the empty vector pCA24N or the plasmid pDinF. In contrast with what happens with NorM from *E. coli* [28] and DinF from *R. solanacearum* [27], our results indicate that expression of the *E. coli dinF* gene in *E. coli* conferred only a slight resistance to bile salts (Table 1). These results suggest that DinF has a narrower substrate spectrum than other multi-drug resistant efflux pumps of the MATE family.

## Discussion

The SOS system is a network that regulates the expression of at least 40 genes, many of them playing key roles in DNA damage tolerance mechanisms, in response to DNA damage [3]. Although the function of most SOS genes is known in *E. coli* and other bacteria [1], there are neither assigned functions nor phenotypes for some others, such as *dinF*. Since it forms a unique transcriptional unit with *lexA,* it would be expected that transcription of *dinF* is tightly regulated by LexA [29]. Consequently, any agent with the potential to induce transcription of *lexA* would, in principle, induce that of *dinF*. Apart from the classic SOS inducers, including UV, mitomycin C and gamma-radiation, increased levels of ROS have been reported to induce the SOS response [30], [31]. To protect all cellular components, including DNA, proteins and lipids, from damage, rapid and coordinated responses are essential for all living organisms. Natural selection has produced a number of systems to prevent or repair DNA damage. Post-replication mismatch repair system (MMR) mainly repairs replication errors [32]. Endogenous DNA damage is primarily repaired by base excision repair (BER) (for a review see reference [1]). Very important are oxidative DNA lesions, which play a major role in spontaneous mutagenesis [33]. Oxidation of guanine to 7,8-dihydro-8-oxoguanine (8-oxoG) is especially noteworthy because, if not repaired, this base lesion can be bypassed by DNA polymerases and originate mutations [34], [35]. In *E. coli* there are specialized proteins, belonging to the so-called GO system, dedicated to alleviate the mutagenicity of 8-oxodGTP [22]. One of these proteins is the MutT enzyme, a nucleoside triphosphate pyrophosphohydrolase, which converts 8-oxodGTP to 8-oxodGMP and pyrophosphate, and inactivates this mutagenic activity. In the absence of MutT there is an increase in AT to CG mutations [22]. Interestingly, *E. coli* and *Pseudomonas aeruginosa mutT*-deficient strains are severely impaired in survival under hydrogen peroxide challenge [36]. MutT also hydrolyzes 8-oxoGTP, preventing incorporation of 8-oxo-Gua into RNA [37]. Recently, NorM, a MATE-family efflux pump, has been demonstrated to protect the cell from the increased hydrogen peroxide killing caused by the lack of MutT [8].

Multi-drug efflux pumps, able to extrude chemicals that can potentially damage DNA, RNA, and proteins, have appeared and been refined through evolution [38]. Therefore, one might speculate that some of these proteins may have also evolved a transcriptional regulation related with the SOS system. However, the expression of none of these proteins is known to be regulated by LexA [3]. According to its deduced polypeptide sequence, DinF is the prototype of a branch of the new family of multidrug and toxin compound extrusion (MATE) membrane proteins [34], [35]. Thus, it is tempting to speculate that DinF could be the first described multidrug efflux pump whose transcription is regulated in response to DNA-damage. In this work we show that expression of DinF is able to reduce the level of intracellular ROS leading, putatively, to the prevention of protein oxidative damage, mutagenesis and the protection from peroxide killing. Despite of the fact that the efflux activity of the *E. coli* DinF protein has still to be proved, our results suggest that DinF may reduce the intracellular pools of potentially oxidizing molecules, diminishing the level of 8-oxodGTP, as suggested by the reduction of the mutation rate of the very specific spectrum of mutations (AT to CG) produced in the *mutT* background.

Cellular extrusion systems in bacteria may protect against toxic compounds like antibiotics and biocides [39], nevertheless, we are far from understanding the real functions due to failing in the identification of its natural substrates. Despite the data obtained with NorM from different bacterial species [15], [26] and DinF from *R. solanacearum* [27], our results suggest that DinF from *E. coli* is not involved in the resistance to antibiotics and other toxics, except bile salts.

Our data from competitions between wild type and the *dinF* mutant, together with data from competitions between wild type and the complemented mutant, clearly show that *dinF* is involved in protection against bile salts. Moreover, the effect of *dinF* expression on H_2_O_2_ viability, intracellular ROS levels, protein carbonylation and mutation rate in the *mutT* background strongly suggest that bile salt protection can be exerted *via* the reduction of oxidative damage. Finally, the putative strict control of *dinF* transcription by LexA, the master repressor of the SOS system, only in bacteria facing bile salts insults, suggests that this association (*lexA-dinF* operon) has evolved to protect these bacteria against this kind of host defenses. This hypothesis is consistent with the fact that exposure of *Salmonella enterica* to bile salts induces the SOS response, indicating the DNA-damaging activity of bile salts [9].

In summary, we describe here for the first time a role for the SOS-gene *dinF*: protection of DNA and proteins from oxidative molecules and reduction of mutation rate when MutT activity is absent (i.e. increased levels of 8-oxo-dGTP). An especially interesting case of bile/pathogen interaction is found in *S. enterica*, which is exposed to bile in the lumen of the mammalian intestine, where concentrations of bile salts range from 0.2 to 2% [40], and in the gall bladder, where much higher concentrations of bile are found [41]. In addition, one of the main pathogens causing cholecystitis is *E. coli* [41], [42].

According to the predicted function of DinF and its ability to reduce the intracellular ROS levels, it is tempting to speculate that the DinF protective activity could be exerted *via* the extrusion of oxidizing molecules. Since hydrogen peroxide diffuses rapidly through membranes, it seems unlikely that DinF might relieve H_2_O_2_ stress by pumping H_2_O_2_ out of the cell. The presence of the *lexA-dinF* operon only in species from the *Enterobacteriaceae* family, together with the bile salts protection, suggests a bile protective role for DinF in this bacterial family. At this stage of the investigation, the exact nature of the DinF activity remains unknown and requires further studies. Because DinF homologues have been found in all three domains of life, including humans [34], [35], it is conceivable that some of them are also involved in ROS protection.
